# Adverse life outcomes associated with adolescent psychotic experiences and depressive symptoms

**DOI:** 10.1007/s00127-018-1496-z

**Published:** 2018-03-19

**Authors:** Jonathan Davies, Sarah Sullivan, Stanley Zammit

**Affiliations:** 1 Centre for Academic Mental Health, School of Social and Community Medicine, University of Bristol, Oakfield House, Oakfield Grove, Clifton, Bristol, BS8 2BN UK; 2 Centre for Academic Primary Care, School of Social and Community Medicine, University of Bristol, 39 Whatley Road, Bristol, BS8 2PS UK; 3 Division of Psychological Medicine and Clinical Neurosciences, University of Cardiff, Hadyn Ellis Building, Maindy Road, Cathays, Cardiff, CF24 4HQ UK

**Keywords:** ALSPAC, Adolescent, Psychosis, Depression, Social

## Abstract

**Purpose:**

To investigate whether psychotic experiences and depressive symptoms at ages 12 and 18 years are associated with adverse life outcomes across a range of functional domains between 16 and 20 years of age.

**Methods:**

Data were gathered from ALSPAC, a UK birth cohort. Individuals were assessed with the semi-structured Psychosis-Like Symptoms Interview and the Short Mood and Feeling Questionnaire at ages 12 and 18 years. Logistic regression was used to explore associations with outcomes in education, occupation, social functioning, substance use (alcohol, cannabis, smoking, and other drugs), and illegal behaviour between the ages of 16 and 20 years. All associations were adjusted for socio-demographic and childhood confounders and for comorbid psychotic experiences or depressive symptoms.

**Results:**

Psychotic experiences and depression at age 12 were associated with poorer educational, occupational, and social outcomes between the ages of 16 and 20; these withstood adjustment for confounding. Depressive symptoms at age 12 were also associated with harmful drinking. Psychotic experiences and depression at age 18 were additionally associated with other forms of substance use and illegal behaviour. Comorbidity had little impact at age 12, but was associated with significantly worse educational, social, and substance use outcomes at age 18.

**Conclusions:**

Adolescent psychotic experiences and depression represent a risk marker for a number of later adverse outcomes, most consistently with education and employment, but also social impairment, harmful drinking, and substance use. This highlights the importance of recognizing adolescent psychopathology, so that support can be provided to try and minimize adverse outcomes.

**Electronic supplementary material:**

The online version of this article (10.1007/s00127-018-1496-z) contains supplementary material, which is available to authorized users.

## Introduction

Psychotic experiences, which are usually regarded as subclinical manifestations of a psychosis continuum [1], and depression are both common during adolescence. Depression becomes increasingly prevalent at this time [2], affecting 8–21% of young people by the age of 18 [2–4]. The prevalence of psychotic experiences, assessed using semi-structured interviews rather than self-report measures, is 5–6% [5–7]. Unlike depression, this prevalence appears to be relatively stable during adolescence [7], with evidence suggesting that they become less common with age [8]. Adolescence represents a period of life when major educational, occupational, and social transitions typically occur, so it is important to determine whether mental health issues arising at this time have a lasting effect on these domains. While there is considerable research on the mental health outcomes of adolescent depression [9–11], and growing research on those of psychotic experiences [7, 12–15], much less is known about their broader psychosocial impact.

Depression during adolescence has been associated with a number of adverse outcomes in later life including poor educational performance, unemployment, lower personal income, welfare dependence, impaired social functioning, delinquent behaviour, smoking, and alcohol and drug abuse [9–11, 16–19]. However, many of these associations have not been consistently replicated across studies, and some associations may not be causal but reflect common antecedent social, familial, and personal factors [10]. Given these ongoing uncertainties about causality, and the fact that findings are based on relatively few cohorts, further investigation of the impact of adolescent depression is needed.

By comparison, even less research has addressed the psychosocial outcomes of adolescent psychotic experiences, and much of what there is comes from cross-sectional studies. Several of these have reported an association with poorer global functioning, both in clinical [20] and community samples [13, 21, 22]. Others have found associations between adolescent psychotic experiences and alcohol use, smoking, cannabis and other drug use, bullying and aggressive behaviour, and school misconduct [23–25]. However, the findings are inconsistent and many of the associations do not survive adjustment for confounding [24]. Studies using longitudinal data have reported that children and young adults with psychotic experiences are more likely to experience worse school performance, behavioural problems, unemployment, interpersonal difficulties, problems with the police and imprisonment [12, 26–28], though none of these studies adjusted for confounding.

In the Avon Longitudinal Study of Parents and Children (ALSPAC) birth cohort, children with psychotic experiences at 12 years of age were more likely to have impaired social functioning at age 13 [29]. While not explained by demographic confounders, the association did not withstand adjustment for comorbid depressive and behavioural symptoms, suggesting that these factors are either confounders or that they lie on the causal pathway from psychotic experiences to poor social functioning. Psychotic experiences and depression are frequently comorbid [6, 13, 14, 30] and share many risk factors in common [31], with some evidence to suggest that they can even be conceptualized as manifestations of a latent common mental distress construct [32]. Indeed, a growing number now view psychotic experiences as a marker of severity in general mental disorder, rather than as a specific forerunner of psychosis [22, 23, 33]. Studies that can jointly investigate the impact of both adolescent depression and psychotic experiences on psychosocial outcomes in adulthood are, therefore, needed to determine whether adverse outcomes associated with these psychopathologies are independent from each other. The current study represents one of the few population-based longitudinal studies with adequate data to address this question. The aims of this study are to:

1. investigate the associations between psychotic experiences and depression in 12 and 18 years with educational, occupational, social, substance use, and illegal and offending behaviour outcomes between the ages of 16 and 20 years of age;
2. examine the extent to which any associations might be explained by confounding;
3. assess whether associations can be accounted for by comorbid psychopathology.

## Methods

### Sample

**Fig. 1:**
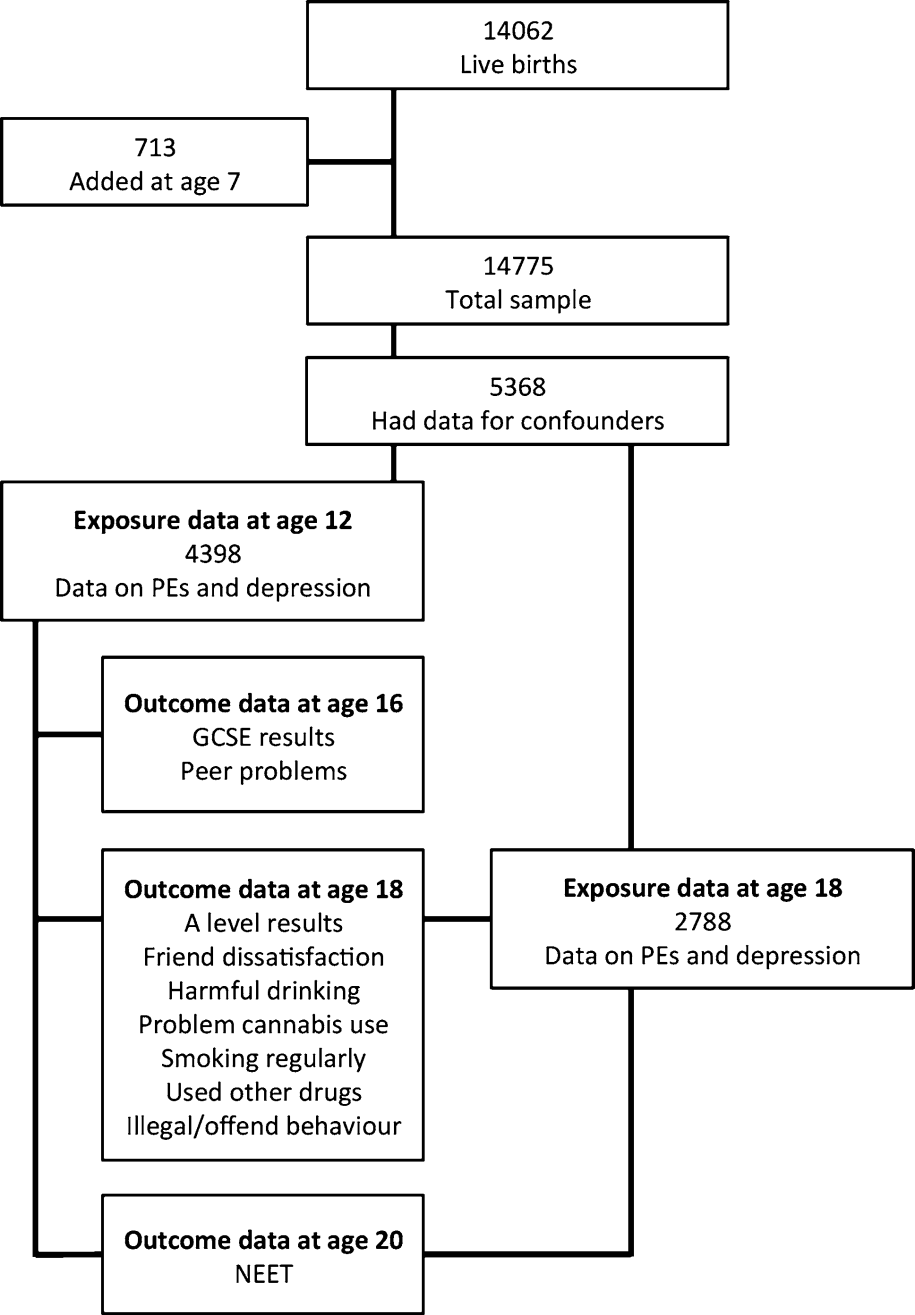
Flow chart of cohort and study participants

The ALSPAC cohort initially consisted of 14,062 children born to residents in the Bristol area, UK, with expected delivery dates between 1st April 1991 and 31st December 1992 (http://www.alspac.bris.ac.uk; fully searchable data dictionary available at http://www.bris.ac.uk/alspac/researchers/data-access/data-dictionary). An additional 713 children who would have been eligible, but were not initially recruited were later identified and enrolled at 7 years of age, resulting in a total sample of 14,775 live births [34]. Young people provided data on psychotic experiences and depressive symptoms at 12 years of age (6796 and 6684 participants, respectively) and at 18 years of age (4720 and 4498 participants, respectively). To be included in the current study, data had to be available for confounding variables, psychotic experiences and depressive symptoms, either at age 12 years (4398 participants) or age 18 years (2788 participants); see Fig. 1. Outcome variables differed in the extent that data were missing meaning analysis sample sizes ranged from 3799 to 2131 participants.

### Exposure measures

#### Psychotic experiences

Psychotic experiences were assessed using the semi-structured Psychosis-Like Symptoms Interview (PLIKSi) [5] at ages 12 and 18 years. The PLIKSi assesses 12 core psychotic experiences over the previous 6 months (for age 12 assessment) or since age 12 (for age 18 assessment): hallucinations (auditory and visual), delusions (spied on, persecution, thoughts read, reference, control, grandiosity, and other unspecified), and experiences of thought interference (broadcasting, insertion, and withdrawal). The interviewer used cross-questioning to establish the presence of symptoms and then rated these as ‘not present’, ‘suspected’, or ‘definitely psychotic’; unclear responses were always rated down. Experiences that the interviewer attributed to sleep or fever have not been coded as psychotic. The current study analysed psychotic experiences in two ways. First, we combined ‘suspected’ and ‘definite’ psychotic experiences into a binary variable (‘not present’ vs. ‘suspected or definite experiences’) to simplify the analyses and increase numbers in each category; such an approach has clinical relevance and is consistent with most literature in the field. A sensitivity analysis preserving the original distinctions was performed, and results were very similar. To examine the impact of severity of psychotic experiences, a ‘psychotic experience score’ was derived from the 12 items on the PLIKSi: responses that were rated as ‘not present’ scored 0, ‘suspected’ scored 1, and ‘definite’ scored 2, providing a total score ranging from 0 to 24.

#### Depressive symptoms

Depressive symptoms were assessed at ages 12 and 18 years via the self-reported Short Mood and Feelings Questionnaire (SMFQ) [35] that covers symptoms experienced over the preceding 2 weeks. Each of its 13 items had 3 possible responses: 0 ‘never’, 1 ‘sometimes’, and 2 ‘always’. This provided a total score marked out of 26 (higher scores indicating more depressive symptoms) that was then used as an exposure variable (referred to as ‘depression score’) for both 12 and 18 years. However, to facilitate comparison with psychotic experiences, a score of 12 or more was used as a cutoff to generate a variable dichotomised between ‘depressed’ and ‘not depressed’. There is no agreed cutoff for the SMFQ, but a precedent for this threshold signifying depression has previously been set [15, 36]; sensitivity analyses using other cutoffs did not substantially alter the results.

### Outcome measures

#### Education and occupation

Educational outcomes were assessed via performance in public examinations, the General Certificate in Secondary Education (GCSE) and the Advanced Level (A level). Information on results was obtained from the National Key Stage 4 and 5 databases, which records data for all pupils in English government maintained schools [34]. GCSEs are typically obtained at age 16 at the end of compulsory schooling in the United Kingdom and are graded A*–G. Participants were dichotomised according to whether they had obtained five or more GCSEs (or equivalent) at grades A*–C, a nationally recognised threshold of attainment. A levels, which are also graded A*–G, are subject-specific qualifications usually taken at age 18. Participants were dichotomised according to whether they had obtained three or more A levels at grades A*–C, the minimum often needed for university entrance, and another nationally recognised threshold of attainment.

Participants were asked about their occupation in a self-reported postal questionnaire sent at age 20 years (average age 20 years 11 months). Those participants who were not in full or part-time education, not in training and were either unemployed or otherwise economically inactive were classified as NEET (not in education, employment or training) in line with the definition used by the Office for National Statistics [37].

#### Social functioning

Social functioning was evaluated through two constructs, peer problems and friend dissatisfaction. The former was assessed using the peer problems subscale of the Strength and Difficulties Questionnaire (SDQ) [38] via a parent-reported postal questionnaire when participants were 16 years. Responses to five statements (each scored 0, 1, or 2) were summed to derive a score from 0 (least problems) to 10 (greatest problems). A cutoff of 4 or more was used to dichotomise participants into those with or without significant peer problems [38, 39]. Friend dissatisfaction is a binary variable that was generated from responses to three items on a self-reported computer questionnaire completed at 18 years. Negative responses (“quite unhappy” or “unhappy”) to questions enquiring about happiness with friendships and satisfaction with number of friends, or scarce endorsement (“not often” or “not at all”) to a question about how often participants felt understood by their friends, were classed as indicative of friend dissatisfaction.

#### Substance use

Information on substance use was collected at 18 years via a self-reported computer questionnaire that included questions on alcohol consumption, cannabis use, smoking frequency, and illicit drug use. The ten-item Alcohol Use Disorders Identification Test (AUDIT) was used to assess alcohol consumption over the past year [40], and a cutoff of 16 or more was used to denote a high level of alcohol problems [41]. Cannabis use in the past 12 months was assessed with the six-item Cannabis Abuse Screen Test (CAST) [42]. Items focus on health, social impairment, and difficulty controlling use, and are scored from 0 (never) to 4 (very often). Participants were classified as having problematic cannabis use if they scored three or more on any of the six items [43]. Information about smoking cigarettes was derived from a series of questions about current smoking frequency. The binary variable of ‘smoking regularly’ was generated according to whether or not participants smoked cigarettes at least weekly, as used in the previous ALPSAC studies [44]. Participants were classified as having used illicit drugs, other than cannabis, if they had indicated using any of the following substances in the past 12 months: cocaine, amphetamines, inhalants, sedatives, hallucinogens, or opioids.

#### Illegal and offending behaviour

Whether participants had been in trouble with the law was assessed through a self-reported computer questionnaire that was completed while they attended the clinic at 18 years. Participants were dichotomised into those that had and those that had not been in trouble during the previous year on the basis of answering ‘yes’ to at least one of the following statements: been in trouble with the police, given a fixed penalty notice, charged for committing a crime, been on trial, received an official police caution, received a fine from Court, given a Community Service Order, given an Antisocial Behaviour Order (ASBO), spent time in a Secure Unit, Young Offender Institution or prison.

### Potential confounders

Based on the literature for psychotic experiences, depression and the outcomes being assessed, the influence of a number of socio-demographic (gender, social class, housing, and maternal education) and childhood (IQ at 8 years, SDQ total score at 8 years) confounders were considered, see Table 1 for details.

**Table 1:** Demographic characteristics of the GCSE sample (*n* = 3799), which is taken to be representative of the overall sample, by psychotic experiences and depression status at 12 years

Variable	*n* (%)	Psychotic experiences at 12 years			Depression at 12 years		
		No (%)	Yes (%)	OR (95% CI)	No (%)	Yes (%)	OR (95% CI)
Gender							
Female	1971 (51.9)	1750 (51.5)	221 (55.1)		1853 (51.2)	118 (64.5)	
Male	1828 (48.1)	1648 (48.5)	180 (44.9)	0.86 (0.70–1.06)	1763 (48.8)	65 (35.5)	0.58 (0.42–0.79)
Social class^a^							
I	592 (15.6)	545 (16.0)	47 (11.7)		563 (15.6)	29 (15.9)	
II	1759 (46.3)	1561 (45.9)	198 (49.4)	1.47 (1.05–2.05)	1672 (46.2)	87 (47.5)	1.01 (0.66–1.55)
III	1316 (34.6)	1175 (34.6)	141 (35.2)	1.39 (0.99–1.97)	1254 (34.7)	62 (33.9)	0.96 (0.61–1.51)
IV–V	132 (3.5)	117 (3.4)	15 (3.7)	1.49 (0.80–2.75)	127 (3.5)	5 (2.7)	0.76 (0.29–2.01)
Housing type							
Mortgaged/owned	3315 (87.3)	2983 (87.8)	332 (82.8)		3161 (87.4)	154 (84.2)	
Council rented	205 (5.4)	171 (5.0)	34 (8.5)	1.79 (1.22–2.63)	189 (5.2)	16 (8.7)	1.74 (1.02–2.97)
Private rented	162 (4.3)	138 (4.1)	24 (6.0)	1.56 (1.00–2.45)	154 (4.3)	8 (4.4)	1.07 (0.51–2.21)
Other	117 (3.1)	106 (3.1)	11 (2.7)	0.93 (0.50–1.75)	112 (3.1)	5 (2.7)	0.92 (0.37–2.28)
Maternal education^b^							
Less than O level	698 (18.4)	619 (18.2)	79 (19.7)		669 (18.5)	29 (15.9)	
O level	1370 (36.1)	1217 (35.8)	153 (38.2)	0.99 (0.74–1.31)	1300 (36.0)	70 (38.3)	1.24 (0.80–1.93)
A level	1093 (28.8)	970 (28.6)	123 (30.7)	0.99 (0.74–1.34)	1035 (28.6)	58 (31.7)	1.29 (0.82–2.04)
Degree or above	638 (16.8)	592 (17.4)	46 (11.5)	0.61 (0.42–0.89)	612 (16.9)	26 (14.2)	0.98 (0.57–1.68)
IQ at 8^c^							
< 100	1261 (33.2)	1111 (32.7)	150 (37.4)		1201 (33.2)	60 (32.8)	
≥ 100	2538 (66.8)	2287 (67.3)	251 (62.6)	0.81 (0.66–1.01)	2415 (66.8)	123 (67.2)	1.02 (0.74–1.40)
SDQ score at 8^c^							
Normal range	3703 (97.6)	3315 (97.6)	388 (96.8)		3527 (97.5)	176 (96.2)	
Abnormal range	96 (2.5)	83 (2.4)	13 (3.2)	1.34 (0.74–2.42)	89 (2.5)	7 (3.8)	1.58 (0.72–3.45)

^a^Highest of either parent, with class I = highest and class V = lowest

^b^Highest educational level achieved

^c^Variable dichotomised only for the purpose of this table

### Statistical analysis

Logistic regression models were used to investigate: (1) longitudinal associations between psychotic experiences and depression at age 12 and outcomes at ages 16–20; (2) longitudinal associations between psychotic experiences and depression at age 18 and NEET at age 20; and (3) cross-sectional associations between psychotic experiences and depression at age 18 and outcomes at age 18 (see Fig. 1). The impact of confounders on the associations was examined by comparing unadjusted estimates with those adjusted for confounders. In addition, mutual adjustments were made using either the depression score or psychotic experience score at the same age, e.g., analyses using psychotic experiences at 12 years as the exposure were adjusted for the depression score at 12, and vice versa. We also examined, as an exposure variable, a measure that combined information on depression and psychotic experiences, coded as: (1) neither psychotic experiences nor depression; (2) depression only; (3) psychotic experiences only; and (4) both psychotic experiences and depression. All analyses were conducted using Stata version 13 (StataCorp LP, USA).

## Results

### Descriptive data

In the total ALSPAC sample that completed the PLIKSi at age 12 years (*n* = 6796), 11.6% had psychotic experiences (suspected or definite). The median psychotic experience score was 0, and scores ranged from 0 to 16. At age 18 years (*n* = 4720), 7.9% had psychotic experiences and the median psychotic experience score was 0, with scores ranging from 0 to 20.

In the total ALSPAC sample that completed the SMFQ at age 12 years (*n* = 6684), 5.3% met the criterion for depression. The mean depression score was 4.0 (standard deviation 3.9). At age 18 years (*n* = 4498), 18.2% met the criterion for depression, and the mean depression score was 6.6 (standard deviation 5.2).

Individuals were more likely to have psychotic experiences at 12 years of age if their parents lived in a property rented from the council, but were less likely to have them if their mother had acquired a degree or if at least one of their parents belonged to the highest social class (see Table 1). Depression at 12 was associated with female gender and living in a council-rented property. Further descriptive information for each outcome according to psychotic experiences and depression at ages 12 and 18 is provided in the Electronic Supplementary Material (Tables ESM1 and ESM2). Our analytical sample was more likely to be female, from a higher social class, living in an owned or mortgaged property and have higher maternal education than the rest of the cohort (see Table ESM3).

### Associations with psychotic experiences

There was strong evidence that psychotic experiences at age 12 were associated with poorer educational attainment at age 16, adverse occupational outcomes, and impaired social functioning. These associations were only slightly attenuated by socio-demographic and childhood confounders (Table 2). Adjusting for depression score had a more substantial effect on estimates, explaining 25–50% of associations with NEET and social functioning, though only weakly altering the association with educational attainment. Psychotic experiences at age 12 were not associated with substance use or illegal behaviour in early adulthood. Analyses using the psychotic experience score at age 12 generated a very similar pattern of associations to those of the binary variable (see Table ESM4), although the association with poorer GCSE exam performance was weaker (OR 1.10, 95% CI 0.99–1.23, *p* = 0.08).

**Table 2:** Odds ratios and 95% confidence intervals for psychosocial outcomes between ages 16–20 in relation with the presence of psychotic experiences (PEs) and depression at age 12

	Model 1^a^		Model 2^b^		Model 3^c^	
	OR (95% CI)	*p* value	OR (95% CI)	*p* value	OR (95% CI)	*p* value
Education and employment						
Did not obtain ≥ 5 GCSEs A*– C (*n* = 3799)						
PEs at 12	1.70 (1.33–2.17)	< 0.001	1.49 (1.11–1.99)	0.007	1.45 (1.08–1.96)	0.015
Depression at 12	1.60 (1.13–2.27)	0.008	1.70 (1.10–2.60)	0.015	1.63 (1.06–2.50)	0.026
Did not obtain ≥ 3 A levels A*–C (*n* = 3052)						
PEs at 12	1.33 (1.03–1.71)	0.028	1.18 (0.89–1.57)	0.243	1.29 (0.97–1.73)	0.085
Depression at 12	1.05 (0.73–1.50)	0.802	1.06 (0.71–1.58)	0.767	1.03 (0.69–1.54)	0.880
NEET at 20 (*n* = 2361)						
PEs at 12	2.09 (1.29–3.38)	0.003	1.90 (1.16–3.10)	0.011	1.54 (0.92–2.58)	0.101
Depression at 12	2.30 (1.23–4.32)	0.009	2.06 (1.08–3.95)	0.029	1.79 (0.92–3.48)	0.087
Social functioning						
Peer problems at 16 (*n* = 3408)						
PEs at 12	1.93 (1.35–2.76)	< 0.001	1.52 (1.05–2.21)	0.028	1.28 (0.86–1.89)	0.221
Depression at 12	2.45 (1.54–3.90)	< 0.001	1.99 (1.22–3.24)	0.006	1.87 (1.14–3.06)	0.014
Friends dissatisfaction at 18 (*n* = 2536)						
PEs at 12	1.42 (0.97–2.09)	0.070	1.36 (0.92–2.00)	0.118	1.13 (0.76–1.69)	0.540
Depression at 12	1.85 (1.14–3.02)	0.014	1.75 (1.07–2.86)	0.026	1.71 (1.04–2.81)	0.036
Substance use						
Harmful drinking at 18 (*n* = 2430)						
PEs at 12	1.17 (0.60–2.28)	0.654	1.13 (0.57–2.22)	0.726	0.87 (0.43–1.74)	0.693
Depression at 12	2.15 (1.01–4.57)	0.046	2.13 (1.00–4.56)	0.051	2.12 (0.98–4.57)	0.056
Problem cannabis use at 18 (*n* = 2602)						
PEs at 12	0.70 (0.32–1.52)	0.367	0.63 (0.29–1.39)	0.252	0.54 (0.24–1.20)	0.128
Depression at 12	1.85 (0.88–3.91)	0.106	1.88 (0.88–4.04)	0.105	2.00 (0.93–4.33)	0.078
Smoking regularly at 18 (*n* = 2627)						
PEs at 12	1.32 (0.95–1.84)	0.093	1.21 (0.87–1.69)	0.260	1.11 (0.79–1.56)	0.559
Depression at 12	1.38 (0.88–2.17)	0.163	1.27 (0.80–2.01)	0.307	1.23 (0.78–1.95)	0.378
Used other drugs at 18 (*n* = 2603)						
PEs at 12	1.21 (0.83–1.76)	0.331	1.24 (0.85–1.82)	0.269	1.13 (0.76–1.67)	0.553
Depression at 12	1.30 (0.78–2.17)	0.322	1.36 (0.81–2.29)	0.247	1.33 (0.79–2.26)	0.283
Illegal and offending behaviour						
I llegal and offending behaviour by 18 (*n* = 2478)						
PEs at 12	0.96 (0.66–1.41)	0.839	0.90 (0.61–1.34)	0.601	0.82 (0.56 (1.23)	0.333
Depression at 12	1.14 (0.69–1.89)	0.610	1.28 (0.75–2.17)	0.364	1.31 (0.77–2.24)	0.318

^a^Model 1: unadjusted

^b^Model 2: adjusted for gender, social class, housing, maternal education, IQ at 8, total SDQ score at 8

^c^Model 3: as for Model 2, and additionally adjusted for either depression score at 12 (for analyses of PEs) or PE score at 12 (for analyses of depression)

There was strong evidence that psychotic experiences at age 18 were associated with negative educational, social, substance use, and illegal behaviour outcomes at the same age, and with poorer occupational outcomes at age 20 (Table 3). These associations were, in general, only slightly attenuated, if at all, by adjustment for socio-demographic and childhood confounders, although the association with NEET was attenuated by approximately 40%. Adjusting for depression score attenuated these associations more substantially, particularly for social functioning. Evidence of association for all outcomes apart from social functioning persisted in these fully adjusted models. Analyses using the psychotic experience score at age 18 yielded a similar pattern of associations (Table ESM5), with stronger evidence of adverse occupational outcomes (OR 1.30, 95% CI 1.06–1.60, *p* = 0.01), but a weaker evidence for A-level performance (OR 1.15, 95% CI 1.00–1.32, *p* = 0.05).

**Table 3:** Odds ratios and 95% confidence intervals for psychosocial outcomes between ages 18–20 in relation with the presence of psychotic experiences (PEs) and depression at age 18

	Model 1^a^		Model 2^b^		Model 3^c^	
	OR (95% CI)	*p* value	OR (95% CI)	*p* value	OR (95% CI)	*p* value
Education and employment						
Did not obtain ≥ 3 A levels A*–C (*n* = 2131)						
PEs at 18	1.93 (1.31–2.83)	0.001	1.91 (1.25–2.93)	0.003	1.63 (1.05–2.51)	0.028
Depression at 18	1.88 (1.46–2.42)	< 0.001	1.90 (1.44–2.51)	< 0.001	1.84 (1.38–2.44)	< 0.001
NEET at 20 (*n* = 1861)						
PEs at 18	2.38 (1.23–4.62)	0.010	1.78 (0.89–3.55)	0.105	1.57 (0.77–3.21)	0.218
Depression at 18	1.94 (1.17–3.24)	0.011	1.57 (0.93–2.67)	0.093	1.45 (0.85–2.49)	0.176
Social functioning						
Friends dissatisfaction at 18 (*n* = 2419)						
PEs at 18	1.89 (1.24–2.89)	0.003	1.82 (1.18–2.79)	0.006	1.08 (0.68–1.71)	0.744
Depression at 18	3.51 (2.64–4.66)	< 0.001	3.48 (2.60–4.66)	< 0.001	3.34 (2.49–4.49)	< 0.001
Substance use						
Harmful drinking at 18 (*n* = 2308)						
PEs at 18	4.60 (2.70–7.82)	< 0.001	4.84 (2.81–8.36)	< 0.001	3.69 (2.08–6.55)	< 0.001
Depression at 18	1.99 (1.20–3.28)	0.007	2.05 (1.23–3.42)	0.006	1.79 (1.05–3.06)	0.031
Problem cannabis use at 18 (*n* = 2475)						
PEs at 18	5.68 (3.48–9.27)	< 0.001	5.79 (3.44–9.72)	< 0.001	3.64 (2.10–6.32)	< 0.001
Depression at 18	3.81 (2.47–5.88)	< 0.001	4.24 (2.69–6.67)	< 0.001	3.55 (2.21–5.68)	< 0.001
Smoking regularly at 18 (*n* = 2491)						
PEs at 18	2.46 (1.73–3.50)	< 0.001	2.19 (1.53–3.15)	< 0.001	1.68 (1.16–2.45)	0.006
Depression at 18	2.44 (1.88–3.17)	< 0.001	2.28 (1.75–2.98)	< 0.001	2.19 (1.67–2.87)	< 0.001
Used other drugs at 18 (*n* = 2475)						
PEs at 18	3.06 (2.13–4.39)	< 0.001	3.41 (2.34–4.98)	< 0.001	2.56 (1.73–3.81)	< 0.001
Depression at 18	2.02 (1.51–2.69)	< 0.001	2.33 (1.73–3.14)	< 0.001	2.05 (1.51–2.79)	< 0.001
Illegal and offending behaviour						
Illegal and offending behaviour by 18 (*n* = 2369)						
PEs at 18	1.99 (1.35–2.93)	0.001	2.19 (1.45–3.30)	< 0.001	1.85 (1.20–2.83)	0.005
Depression at 18	1.30 (0.96–1.75)	0.089	1.43 (1.04–1.96)	0.026	1.33 (0.96–1.84)	0.083

^a^Model 1: unadjusted

^b^Model 2: adjusted for gender, social class, housing, maternal education, IQ at 8, total SDQ score at 8

^c^Model 3: as for Model 2, and additionally adjusted for either depression score at 18 (for analyses of PEs) or PE score at 18 (for analyses of depression)

### Associations with depression

Depression at age 12 was associated with poorer educational, occupational and social outcomes at ages 16–20 years (Table 2). Adjusting for socio-demographic and childhood confounders generally resulted in minimal change to these associations, as did adjustments for psychotic experience score. However, results for analyses using the depression score at age 12 were not entirely consistent (Table ESM4). Depression score showed no association with educational attainment at age 16 (OR 1.02, 95% CI 0.99–1.04, *p* = 0.21), while there was weak evidence that higher depression scores were associated with better educational outcomes age 18 (OR 1.15, 95% CI 1.00–1.32, *p* = 0.05). Furthermore, while there was no evidence that the dichotomised depression variable was associated with substance use, there was strong evidence that depression score was associated with these outcomes (harmful drinking *p* < 0.001; regular smoking *p* = 0.01; other drug use *p* = 0.02), albeit weakly so for problem cannabis use (*p* = 0.05).

The picture was more consistent for depression at 18 years. There was robust evidence that depression at 18 was associated with negative educational, social, substance use, and illegal behaviour outcomes at that age, and with adverse occupational outcomes at age 20 (Table 3). Many associations were slightly strengthened by adjustments for socio-demographic and childhood confounders, with the remaining associations only weakly attenuated. The exception to this was NEET at age 20, which was attenuated by almost 40%. Adjusting for psychotic experience score attenuated most associations by a small degree, with more substantial attenuation for those associations with problem cannabis use and other drug use. Analyses using the depression score at age 18 generated a very similar pattern of associations (Table ESM5).

### Associations with comorbid psychotic experiences and depression

Having comorbid psychotic experiences and depression at age 12 was not associated with poorer outcomes in early adulthood when compared with having either condition alone (Figure ESM1, Table ESM6). The exception to this was friend dissatisfaction at 18, where there was weak evidence that only having psychotic experiences was associated with lower risk when compared with having both psychotic experiences and depression (OR 0.43, 95% CI 0.17–1.05, *p* = 0.06).

**Fig. 2:**
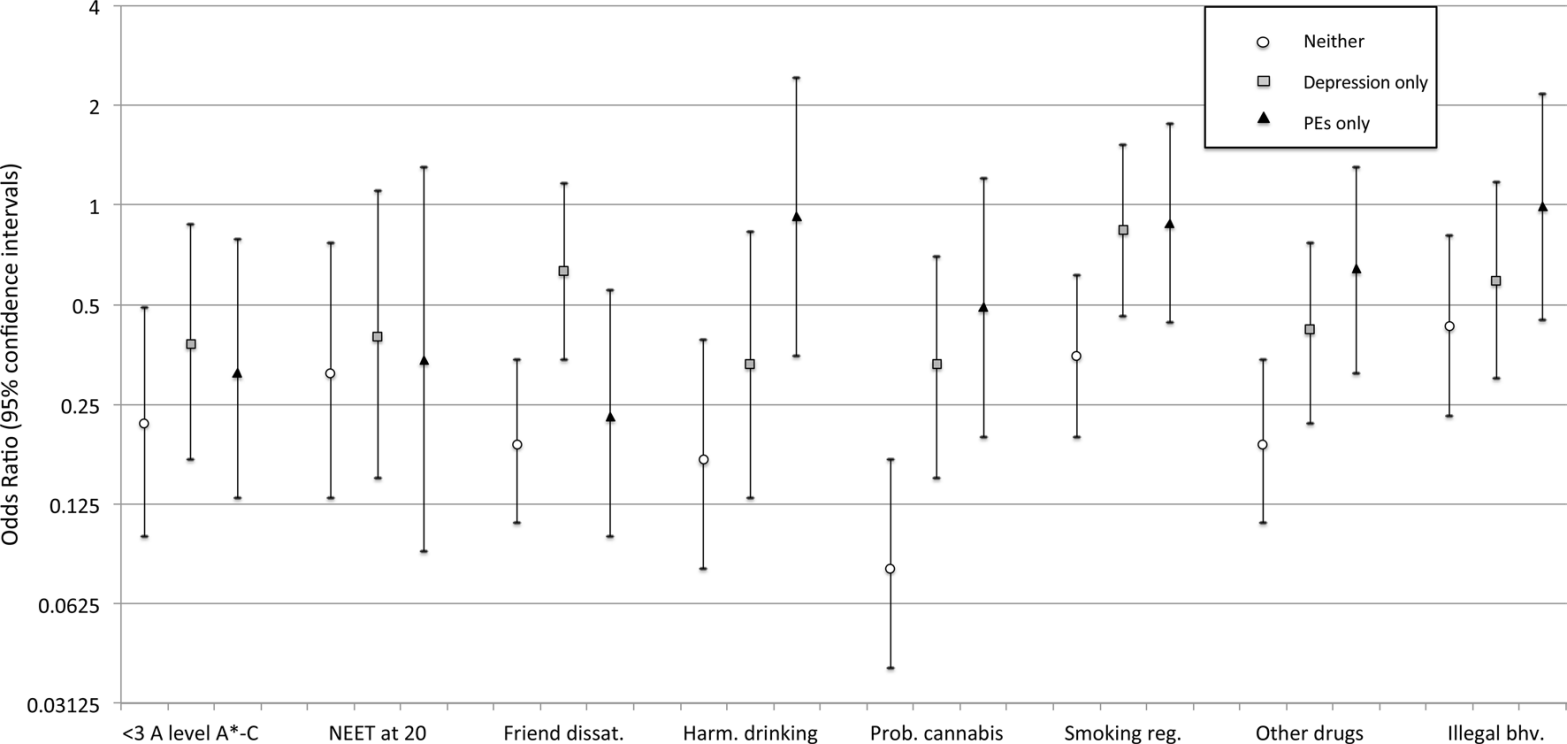
Odds ratios and 95% confidence intervals for psychosocial outcomes between ages 18–20 in relation with the presence of ‘neither depression nor PEs’, ‘PEs only’, and ‘depression only’ at 18 years. Having ‘both PEs and depression’ was used as the baseline reference group, meaning that OR < 1 indicates having ‘both’ was detrimental. Associations have been adjusted for confounders (gender, social class, housing, maternal education, IQ at 8, and total SDQ score at 8)

At age 18, the negative impact of comorbidity was more evident (Fig. 2, Table ESM7). Individuals were less likely to have poor A-level attainment if they only had depression or only psychotic experiences when compared with those who had both. In comparison with comorbid individuals, only having depression was associated with a lower risk of harmful alcohol use, problem cannabis use and other drug use at age 18, and weakly associated with less chance of being NEET at age 20. There was also lower risk of friend dissatisfaction at age 18 for individuals who only had psychotic experiences compared with those who were comorbid with depression.

## Discussion

Having psychotic experiences or depression at 12 years of age was associated with adverse life outcomes in early adulthood. Both were associated with poorer educational performance at age 16, and affected individuals were about twice as likely to be NEET at age 20. Social functioning was also impaired, but the association with psychotic experiences was primarily driven by comorbid depressive symptoms, which were either confounding the association or lying on the causal pathway between psychotic experiences and social dysfunction. These competing explanations of psychotic experience-associated social impairment have been raised before [29], but it was not possible to tease them out here. In contrast, the relationship with poor GCSE performance was relatively independent of comorbid symptoms, suggesting that its association with psychotic experiences and depression was mediated differently to that of social functioning. For example, both psychotic experiences and depression could have independent effects on concentration that impair academic performance, whereas the key impact on social functioning might be through a characteristic that is more central to depression, such as low self-esteem.

The evidence of associations at age 12 was generally stronger for the depression score than the binary depression threshold measure. This was as expected given the former offers a more powerful approach, and is consistent with effects being present across the spectrum of depressive symptomatology. In comparison, this was much less evident for the psychotic experience score, perhaps suggesting that the presence of any psychotic experience indicates psychopathology that is sufficiently severe to explain associations with adverse outcomes. Thus, the psychotic experience score might not be assessing the spectrum of sub-threshold effects as the SMFQ does for depression.

The relationship between depressive symptoms at age 12 and educational performance was not straightforward. Unlike the binary variable, the depression score was not associated with worse educational attainment at age 16. Indeed, there was evidence of a weakly positive effect on educational attainment at age 18. This might have been a chance finding, but it could be that the SMFQ was measuring something more than depressive symptoms. Perhaps, it also detected personality traits like neuroticism and introversion that, in moderation, could be associated with better educational performance. However, a positive association was not replicated for depression score at age 18, which potentially undermines this explanation. In fact, depression score at age 18 was associated with worse education outcomes at this age. It could be that the SMFQ measures depression to a greater extent than neuroticism at age 18, but to a lesser extent at age 12; the higher SMFQ mean score and the proportion meeting criteria for depression at this age would be consistent with this account.

By age 18, psychotic experiences were less prevalent than depression, a reversal of the picture seen at age 12. There was strong evidence of associations between psychotic experiences and depression with poorer educational outcomes, harmful drinking, problem cannabis use, smoking, and use of other drugs. Psychotic experiences and depression were additionally associated with an increased likelihood of illegal behaviour, although the association with depression seems to have been largely attributable to comorbid psychotic experiences. The reverse was true for friend dissatisfaction, which was underpinned by comorbid depressive symptoms rather than psychotic experiences.

Aside from friend dissatisfaction, there was little to suggest that comorbid psychotic experiences and depression at age 12 were associated with poorer outcomes than with having either condition alone. In contrast, comorbidity had significantly more negative impact at age 18, particularly when compared with the educational and substance use outcomes of only having depression, supporting the thesis that psychotic experiences are an index of mental disorder severity [32].

Taken collectively, these findings suggest that adolescent psychotic experiences are not as benign as those limited to childhood [27, 28]. Even discounting the minority who go on to develop mental health problems [7, 12–14], the lives of many individuals may be negatively affected in other ways for a considerable period of time. The association of psychotic experiences, both at ages 12 and 18, with poorer educational and occupational outcomes is consistent with findings in other longitudinal studies [12, 26]. This is also true of the association with social impairment, although this seems to have been driven by comorbid depressive symptoms, as has been previously found in the ALSPAC cohort [29]. Other adverse outcomes, particularly those involving antisocial behaviour (as indexed by our measure of illegal and offending behaviour), drug use and alcohol abuse, were only associated with psychotic experiences at age 18. These are cross-sectional analyses, so it is not possible to comment on the direction of causality, but they are consistent with associations described elsewhere [23–26]. These additional adverse associations may also be a function, at least in part, of the time period being described: questions about psychotic experiences at age 12 covered the preceding 6 months compared with 6 years at age 18. Thus, the latter may have captured more chronic presentations.

The results for adolescent depression were broadly consistent with associations previously reported with impaired social functioning [9, 16] and negative educational and employment outcomes [10, 11, 16, 17]. Alcohol abuse, which has been one of the most consistent associations in the past research [11, 18, 19], was the substance-related issue most strongly associated with depression at age 12. Earlier findings that adolescent depression is associated with smoking, illicit drug use, and delinquent behaviour [11, 16, 19] were much more evident in the associations with depression at age 18. Adjusting for confounders did not unduly diminish the associations found here, unlike in the Christchurch cohort where associations between adolescent depression and adverse education and employment outcomes, nicotine dependence and alcohol abuse were eliminated following adjustment [10]. While we adjusted for many of the same covariates (e.g., maternal education, social class, and IQ), we were not able to adjust for others such as childhood sexual abuse and affiliating with substance-abusing peers. Thus, residual confounding could exist within the present study.

The current study has a number of strengths including: a longitudinal design with reasonably long follow-up, access to a range of psychosocial measures, including some that are interviewer-rated (e.g., the PLIKSi), and use of objective national exam data sets. It also has its limitations. Despite adjusting for a reasonably comprehensive set of confounders, residual confounding remains possible. Furthermore, as most of the associations with psychotic experiences and depression at age 18 are cross section, it is possible that psychopathology resulted as a consequence of the poorer psychosocial outcomes. Despite the large sample size, the relatively rare nature of the exposures and outcomes often meant that the analyses were likely to be underpowered, and the confidence intervals were often wide. There was also considerable attrition in the ALSPAC cohort that could have introduced selection bias. This has been explored elsewhere [34], but simulation studies in ALSPAC have shown that while prevalence estimates are under-estimated, associations are only marginally affected by selective attrition [45]. Nevertheless, it is possible that estimates we report are influenced by attrition bias. A further limitation was that it was not possible to infer whether an individual lacking GCSE or A-level data were the result of him or her failing these or not having sat the exam, because data from private schools were not included in the data sets. This meant that students not progressing onto these exams, perhaps because of their psychotic experiences or depression, would not have been included in our analyses, potentially underestimating associations with adverse educational outcomes.

In light of these findings, it is important that adolescent psychotic experiences and depressive symptoms are identified as early as possible because they represent a risk marker for a number of adverse outcomes in later life, most consistently with education and employment. Current UK guidelines for managing adolescent psychosis recommend additional educational support when performance has been affected and advocate the provision of supported employment programmes and work-related activities [46]. The depression guidelines are less explicit, but do identify the need to address educational problems [47]. Meanwhile, the strong links with alcohol, cannabis, and other drug use for older teenagers reporting psychotic experiences and depression, while not necessarily causal, highlight the need to provide psychoeducation and support. Future research needs to build on the limited evidence, we currently have about long-term functional outcomes. One way would be to construct more sophisticated models to test whether variables like drug and alcohol misuse could be mediating outcomes like unemployment.

## Electronic supplementary material

Below is the link to the electronic supplementary material.

Supplementary material 1 (DOCX 61 KB)

Supplementary material 1 (DOCX 402 KB)
