# Supplementary material for: Adverse life outcomes associated with adolescent psychotic experiences and depressive symptoms
Source: Soc Psychiatry Psychiatr Epidemiol. 2018 Mar 19;53(5):497–507. doi: 10.1007/s00127-018-1496-z (PMC5908822; doi:10.1007/s00127-018-1496-z)
Supplement: Supplementary file 1 — Supplementary material 1 (DOCX 61 KB) [file 127_2018_1496_MOESM1_ESM.docx]

**ELECTRONIC SUPPLEMENTARY MATERIAL (ESM)**

*Please refer to attachment for figure*

**Fig. ESM1** Odds ratios and 95% confidence intervals for psychosocial outcomes between ages 16–20 in relation to the presence of ‘neither depression nor PEs’, ‘PEs only’ and ‘depression only’ at 12 years. Having ‘both PEs and depression’ was used as the baseline reference group, meaning OR < 1 indicates having ‘both’ was detrimental. Associations have been adjusted for confounders (gender, social class, housing, maternal education, IQ at 8, total SDQ score at 8)

**Table ESM1** Frequencies of outcomes in early adulthood (16 to 20 years) according to the presence of psychotic experiences or depression at age 12

|  | | ***n (%)*** | **Psychotic experiences at 12 years** | | **Depression at 12 years** | |
| --- | --- | --- | --- | --- | --- | --- |
|  |  |  | **No *n* (%)** | **Yes *n* (%)** | **No *n* (%)** | **Yes *n* (%)** |
| **Education & employment** |  |  |  |  |  |  |
| Obtained ≥ 5 GCSEs A*– C | Yes | 3143 (82.7) | 2842 (83.6) | 301 (75.1) | 3005 (83.1) | 138 (75.4) |
|  | No | 656 (17.3) | 556 (16.4) | 100 (24.9) | 611 (16.9) | 45 (24.6) |
| Obtained ≥ 3 A levels A*– C | Yes | 1202 (39.4) | 1103 (40.0) | 99 (33.5) | 1151 (39.4) | 51 (38.4) |
|  | No | 1850 (60.6) | 1653 (60.0) | 197 (66.6) | 1768 (60.6) | 82 (61.7) |
| NEET at 20 | No | 2233 (94.6) | 2031 (95.0) | 202 (90.2) | 2137 (94.9) | 96 (88.9) |
|  | Yes | 128 (5.4) | 106 (5.0) | 22 (9.8) | 116 (5.2) | 12 (11.1) |
| **Social functioning** |  |  |  |  |  |  |
| Peer problems at 16 | No | 3171 (93.1) | 2861 (93.6) | 310 (88.3) | 3038 (93.4) | 133 (85.3) |
|  | Yes | 237 (7.0) | 196 (6.4) | 41 (11.7) | 214 (6.6) | 23 (14.7) |
| Friends dissatisfaction at 18 | No | 2262 (89.2) | 2051 (89.6) | 211 (85.8) | 2165 (89.5) | 97 (82.2) |
|  | Yes | 274 (10.8) | 239 (10.4) | 35 (14.2) | 253 (10.5) | 21 (17.8) |
| **Substance use** |  |  |  |  |  |  |
| Harmful drinking at 18 | No | 2342 (96.4) | 2110 (96.4) | 232 (96.0) | 2238 (96.6) | 104 (92.9) |
|  | Yes | 88 (3.6) | 78 (3.6) | 10 (4.1) | 80 (3.5) | 8 (7.1) |
| Problem cannabis use at 18 | No | 2505 (96.3) | 2254 (96.2) | 251 (97.3) | 2389 (96.4) | 116 (93.6) |
|  | Yes | 97 (3.7) | 90 (3.8) | 7 (2.7) | 89 (3.6) | 8 (6.5) |
| Smoking regularly at 18 | No | 2218 (84.4) | 2007 (84.8) | 211 (80.8) | 2118 (84.7) | 100 (80.0) |
|  | Yes | 409 (15.6) | 359 (15.2) | 50 (19.2) | 384 (15.4) | 25 (20.0) |
| Used other drugs at 18 | No | 2298 (88.3) | 2075 (88.5) | 223 (86.4) | 2192 (88.4) | 106 (85.5) |
|  | Yes | 305 (11.7) | 270 (11.5) | 35 (13.6) | 287 (11.6) | 18 (14.5) |
| **Illegal and offending behaviour** | | |  |  |  |  |
| Illegal and offending behaviour by 18 | No | 2119 (85.5) | 1911 (85.5) | 208 (86.0) | 2020 (85.6) | 99 (83.9) |
|  | Yes | 359 (14.5) | 325 (14.5) | 34 (14.1) | 340 (14.4) | 19 (16.1) |

**Table ESM2** Frequencies of outcomes in early adulthood (18 to 20 years) according to the presence of psychotic experiences or depression at age 18

|  | | ***n (%)*** | **Psychotic experiences at 18 years** | | **Depression at 18 years** | |
| --- | --- | --- | --- | --- | --- | --- |
|  |  |  | **No *n* (%)** | **Yes *n* (%)** | **No *n* (%)** | **Yes *n* (%)** |
| **Education & employment** |  |  |  |  |  |  |
| Obtained ≥ 3 A levels A*– C | Yes | 933 (43.8) | 894 (44.7) | 39 (29.6) | 831 (46.1) | 102 (31.2) |
|  | No | 1198 (56.2) | 1105 (55.3) | 93 (70.5) | 973 (53.9) | 225 (68.8) |
| NEET at 20 | No | 1775 (95.4) | 1672 (95.7) | 103 (90.4) | 1522 (95.9) | 253 (92.3) |
|  | Yes | 86 (4.6) | 75 (4.3) | 11 (9.7) | 65 (4.1) | 21 (7.7) |
| **Social functioning** |  |  |  |  |  |  |
| Friends dissatisfaction at 18 | No | 2162 (89.4) | 2026 (89.9) | 136 (82.4) | 1870 (91.9) | 292 (76.2) |
|  | Yes | 257 (10.6) | 228 (10.1) | 29 (17.6) | 166 (8.2) | 91 (23.8) |
| **Substance use** |  |  |  |  |  |  |
| Harmful drinking at 18 | No | 2226 (96.5) | 2080 (97.1) | 146 (88.0) | 1879 (96.9) | 347 (94.0) |
|  | Yes | 82 (3.6) | 62 (2.9) | 20 (12.1) | 60 (3.1) | 22 (6.0) |
| Problem cannabis use at 18 | No | 2384 (96.3) | 2235 (97.1) | 149 (85.6) | 2021 (97.4) | 363 (90.8) |
|  | Yes | 91 (3.7) | 66 (2.9) | 25 (14.4) | 54 (2.6) | 37 (9.3) |
| Smoking regularly at 18 | No | 2132 (85.6) | 2006 (86.6) | 126 (72.4) | 1834 (87.7) | 298 (74.5) |
|  | Yes | 359 (14.4) | 311 (13.4) | 48 (27.6) | 257 (12.3) | 102 (25.5) |
| Used other drugs at 18 | No | 2187 (88.4) | 2059 (89.5) | 128 (73.6) | 1862 (89.7) | 325 (81.3) |
|  | Yes | 288 (11.6) | 242 (10.5) | 46 (26.4) | 213 (10.3) | 75 (18.8) |
| **Illegal and offending behaviour** | | |  |  |  |  |
| Illegal and offending behaviour by 18 | No | 2046 (86.4) | 1921 (987.0) | 125 (77.2) | 1724 (86.9) | 322 (83.6) |
|  | Yes | 323 (13.6) | 286 (13.0) | 37 (22.8) | 260 (13.1) | 63 (16.4) |

**Table ESM3** Demographic characteristics of the total sample compared with the analytical sample (based here on the GCSE sample, *n* = 3799, which is taken to be representative of other analyses).

| **Variable** | **Total ALSPAC sample** | | **Analytical sample** | |
| --- | --- | --- | --- | --- |
|  |  | ***n* (%)** |  | ***n* (%)** |
| **Gender** |  |  |  |  |
| Female |  | 6770 (49.0) |  | 1971 (51.9) |
| Male |  | 7059 (51.0) |  | 1828 (48.1) |
| **Social class^a^** |  |  |  |  |
| I |  | 1543 (13.3) |  | 592 (15.6) |
| II |  | 4843 (41.8) |  | 1759 (46.3) |
| III |  | 4523 (39.0) |  | 1316 (34.6) |
| IV – V |  | 686 (5.9) |  | 132 (3.5) |
| **Housing type** |  |  |  |  |
| Mortgaged/owned |  | 9894 (73.1) |  | 3315 (87.3) |
| Council rented |  | 1946 (14.4) |  | 205 (5.4) |
| Private rented |  | 987 (7.3) |  | 162 (4.3) |
| Other |  | 703 (5.2) |  | 117 (3.1) |
| **Maternal education^b^** |  |  |  |  |
| Less than O level |  | 3761 (30.1) |  | 698 (18.4) |
| O level |  | 4334 (34.6) |  | 1370 (36.1) |
| A level |  | 2805 (22.4) |  | 1093 (28.8) |
| Degree or above |  | 1612 (12.9) |  | 638 (16.8) |

**^a^** Highest of either parent, with class I = highest and class V = lowest

**^b^** Highest educational level achieved

^c^ Variable dichotomised only for the purpose of this table

**Table ESM4** Odds ratios and 95% confidence intervals for psychosocial outcomes between ages 16–20 in relation to the psychotic experience (PE) score and depression score at age 12

|  | | **Model 1^a^** | | **Model 2^b^** | | **Model 3^c^** | |
| --- | --- | --- | --- | --- | --- | --- | --- |
|  |  | **OR (95% CI)** | **P val** | **OR (95% CI)** | **P val** | **OR (95% CI)** | **P val** |
| **Education & employment** | | | | | | | |
| Did not obtain ≥ 5 GCSEs A*– C (*n* = 3799) | | | | | | | |
|  | PE score at 12 | 1.14 (1.04 to 1.24) | 0.005 | 1.10 (0.99 to 1.23) | 0.078 | 1.09 (0.98 to 1.22) | 0.127 |
|  | Depression score at 12 | 1.01 (0.99 to 1.04) | 0.262 | 1.02 (0.99 to 1.04) | 0.207 | 1.01 (0.99 to 1.04) | 0.356 |
| Did not obtain ≥ 3 A levels A*– C (*n* = 3052) | | | | | | | |
|  | PE score at 12 | 1.12 (1.01 to 1.25) | 0.028 | 1.08 (0.96 to 1.21) | 0.193 | 1.11 (0.99 to 1.25) | 0.072 |
|  | Depression score at 12 | 0.97 (0.95 to 0.99) | 0.006 | 0.98 (0.95 to 1.00) | 0.034 | 0.97 (0.95 to 0.99) | 0.014 |
| NEET at 20 (*n* = 2361) | | | | | | | |
|  | PE score at 12 | 1.31 (1.12 to 1.53) | 0.001 | 1.28 (1.09 to 1.51) | 0.002 | 1.21 (1.02 to 1.44) | 0.026 |
|  | Depression score at 12 | 1.08 (1.04 to 1.13) | <0.001 | 1.08 (1.04 to 1.13) | <0.001 | 1.07 (1.02 to 1.12) | 0.002 |
| **Social functioning** | | | | | | | |
| Peer problems at 16 (*n* = 3408) | | | | | | | |
|  | PE score at 12 | 1.23 (1.09 to 1.39) | 0.001 | 1.14 (1.00 to 1.30) | 0.046 | 1.08 (0.95 to 1.24) | 0.248 |
|  | Depression score at 12 | 1.08 (1.05 to 1.12) | <0.001 | 1.06 (1.03 to 1.10) | <0.001 | 1.06 (1.02 to 1.09) | 0.001 |
| Friends dissatisfaction at 18 (*n* = 2536) | | | | | | | |
|  | PE score at 12 | 1.09 (0.95 to 1.26) | 0.230 | 1.08 (0.93 to 1.25) | 0.331 | 1.00 (0.86 to 1.18) | 0.953 |
|  | Depression score at 12 | 1.07 (1.04 to 1.11) | <0.001 | 1.07 (1.04 to 1.10) | <0.001 | 1.07 (1.04 to 1.10) | <0.001 |
| **Substance use** | | | | | | | |
| Harmful drinking at 18 (*n* = 2430) | | | | | | | |
|  | PE score at 12 | 1.06 (0.82 to 1.36) | 0.663 | 1.05 (0.81 to 1.35) | 0.718 | 0.95 (0.71 to 1.26) | 0.718 |
|  | Depression score at 12 | 1.09 (1.04 to 1.14) | <0.001 | 1.10 (1.05 to 1.15) | <0.001 | 1.10 (1.05 to 1.15) | <0.001 |
| Problem cannabis use at 18 (*n* = 2602) | | | | | | | |
|  | PE score at 12 | 0.89 (0.63 to 1.25) | 0.495 | 0.86 (0.60 to 1.22) | 0.390 | 0.79 (0.55 to 1.15) | 0.225 |
|  | Depression score at 12 | 1.04 (1.00 to 1.10) | 0.074 | 1.05 (1.00 to 1.10) | 0.049 | 1.06 (1.01 to 1.11) | 0.026 |
| Smoking regularly at 18 (*n* = 2627) | | | | | | | |
|  | PE score at 12 | 1.10 (0.97 to 1.25) | 0.138 | 1.08 (0.95 to 1.22) | 0.269 | 1.04 (0.91 to 1.19) | 0.564 |
|  | Depression score at 12 | 1.04 (1.01 to 1.07) | 0.004 | 1.03 (1.01 to 1.06) | 0.013 | 1.03 (1.00 to 1.06) | 0.022 |
| Used other drugs at 18 (*n* = 2603) | | | | | | | |
|  | PE score at 12 | 1.04 (0.90 to 1.21) | 0.589 | 1.05 (0.91 to 1.22) | 0.489 | 1.02 (0.87 to 1.19) | 0.845 |
|  | Depression score at 12 | 1.04 (1.01 to 1.07) | 0.021 | 1.04 (1.01 to 1.07) | 0.020 | 1.04 (1.00 to 1.07) | 0.026 |
| **Illegal and offending behaviour** | | | | | | | |
| Illegal and offending behaviour by 18 (*n* = 2478) | | | | | | | |
|  | PE score at 12 | 0.97 (0.83 to 1.14) | 0.719 | 0.95 (0.81 to 1.12) | 0.530 | 0.91 (0.77 to 1.09) | 0.305 |
|  | Depression score at 12 | 1.01 (0.98 to 1.04) | 0.441 | 1.03 (1.00 to 1.06) | 0.053 | 1.04 (1.00 to 1.07) | 0.034 |

^a^Model 1: unadjusted

^b^Model 2: adjusted for gender, social class, housing, maternal education, IQ at 8, total SDQ score at 8

^c^Model 3: as for Model 2, and additionally adjusted for either depression score at 12 (for analyses of PE score) or PE score at 12 (for analyses of depression score)

**Table ESM5** Odds ratios and 95% confidence intervals for psychosocial outcomes between ages 18–20 in relation to the psychotic experience (PE) score and depression score at age 18

|  | | **Model 1^a^** | | **Model 2^b^** | | **Model 3^c^** | |
| --- | --- | --- | --- | --- | --- | --- | --- |
|  |  | **OR (95% CI)** | **P val** | **OR (95% CI)** | **P val** | **OR (95% CI)** | **P val** |
| **Education & employment** | | | | | | | |
| Did not obtain ≥ 3 A levels A*– C (*n* = 2131) | | | | | | | |
|  | PE score at 18 | 1.16 (1.01 to 1.32) | 0.031 | 1.15 (1.00 to 1.32) | 0.054 | 1.08 (0.94 to 1.24) | 0.284 |
|  | Depression score at 18 | 1.04 (1.02 to 1.06) | <0.001 | 1.04 (1.02 to 1.06) | <0.001 | 1.04 (1.02 to 1.06) | <0.001 |
| NEET at 20 (*n* = 1861) | | | | | | | |
|  | PE score at 18 | 1.38 (1.13 to 1.67) | 0.001 | 1.30 (1.06 to 1.60) | 0.012 | 1.26 (1.02 to 1.56) | 0.030 |
|  | Depression score at 18 | 1.05 (1.01 to 1.09) | 0.011 | 1.04 (1.00 to 1.08) | 0.085 | 1.03 (0.99 to 1.07) | 0.188 |
| **Social functioning** | | | | | | | |
| Friends dissatisfaction at 18 (*n* = 2419) | | | | | | | |
|  | PE score at 18 | 1.21 (1.08 to 1.35) | 0.001 | 1.20 (1.07 to 1.34) | 0.002 | 1.05 (0.93 to 1.18) | 0.431 |
|  | Depression score at 18 | 1.13 (1.11 to 1.16) | <0.001 | 1.13 (1.11 to 1.16) | <0.001 | 1.13 (1.11 to 1.16) | <0.001 |
| **Substance use** | | | | | | | |
| Harmful drinking at 18 (*n* = 2308) | | | | | | | |
|  | PE score at 18 | 1.26 (1.10 to 1.43) | 0.001 | 1.26 (1.10 to 1.44) | 0.001 | 1.17 (1.02 to 1.35) | 0.030 |
|  | Depression score at 18 | 1.08 (1.04 to 1.12) | <0.001 | 1.09 (1.05 to 1.13) | <0.001 | 1.08 (1.04 to 1.12) | <0.001 |
| Problem cannabis use at 18 (*n* = 2475) | | | | | | | |
|  | PE score at 18 | 1.40 (1.24 to 1.58) | <0.001 | 1.42 (1.25 to 1.61) | <0.001 | 1.28 (1.12 to 1.46) | <0.001 |
|  | Depression score at 18 | 1.22 (1.09 to 1.16) | <0.001 | 1.14 (1.10 to 1.18) | <0.001 | 1.12 (1.08 to 1.16) | <0.001 |
| Smoking regularly at 18 (*n* = 2491) | | | | | | | |
|  | PE score at 18 | 1.19 (1.08 to 1.32) | 0.001 | 1.16 (1.05 to 1.29) | 0.004 | 1.08 (0.97 to 1.20) | 0.153 |
|  | Depression score at 18 | 1.08 (1.06 to 1.10) | <0.001 | 1.08 (1.05 to 1.10) | <0.001 | 1.07 (1.05 to 1.10) | <0.001 |
| Used other drugs at 18 (*n* = 2475) | | | | | | | |
|  | PE score at 18 | 1.32 (1.19 to 1.46) | <0.001 | 1.36 (1.22 to 1.51) | <0.001 | 1.26 (1.12 to 1.41) | <0.001 |
|  | Depression score at 18 | 1.07 (1.05 to 1.10) | <0.001 | 1.09 (1.06 to 1.11) | <0.001 | 1.08 (1.05 to 1.10) | <0.001 |
| **Illegal and offending behaviour** | | | | | | | |
| Illegal and offending behaviour by 18 (*n* = 2369) | | | | | | | |
|  | PE score at 18 | 1.15 (1.04 to 1.29) | 0.009 | 1.19 (1.06 to 1.33) | 0.003 | 1.14 (1.01 to 1.28) | 0.037 |
|  | Depression score at 18 | 1.03 (1.01 to 1.05) | 0.008 | 1.05 (1.02 to 1.07) | <0.001 | 1.04 (1.02 to 1.07) | 0.001 |

^a^Model 1: unadjusted

^b^Model 2: adjusted for gender, social class, housing, maternal education, IQ at 8, total SDQ score at 8

^c^Model 3: as for Model 2, and additionally adjusted for either depression score at 18 (for analyses of PE score) or PE score at 18 (for analyses of depression score)

**Table ESM6** Four level analysis of the associations of ‘psychotic experiences only’, ‘depression only’ and ‘both psychotic experiences and depression’ at 12 years (‘neither’ being used as the baseline reference group) with outcomes in early adulthood (16 to 20 years)

|  | | **Model 1^a^** | | **Model 2^b^** | |
| --- | --- | --- | --- | --- | --- |
|  |  | **OR (95% CI)** | **P val** | **OR (95% CI)** | **P val** |
| **Education & employment** | | | | | |
| Did not obtain ≥ 5 GCSEs A*– C (*n* = 3799) | | | | | |
|  | Depression only | 1.64 (1.08 to 2.48) | 0.020 | 1.59 (0.96 to 2.66) | 0.074 |
|  | PEs only | 1.71 (1.32 to 2.23) | <0.001 | 1.43 (1.05 to 1.96) | 0.023 |
|  | Both | 1.88 (1.01 to 3.48) | 0.046 | 2.25 (1.08 to 4.68) | 0.030 |
| Did not obtain ≥ 3 A levels A*– C (*n* = 3052) | | | | | |
|  | Depression only | 0.99 (0.66 to 1.50) | 0.970 | 0.99 (0.63 to 1.57) | 0.975 |
|  | PEs only | 1.33 (1.01 to 1.74) | 0.040 | 1.16 (0.86 to 1.57) | 0.329 |
|  | Both | 1.33 (0.66 to 2.68) | 0.418 | 1.34 (0.63 to 2.84) | 0.449 |
| NEET at 20 (*n* = 2361) | | | | | |
|  | Depression only | 2.32 (1.09 to 4.96) | 0.030 | 2.13 (0.97 to 4.69) | 0.060 |
|  | PEs only | 2.06 (1.22 to 3.48) | 0.007 | 1.90 (1.11 to 3.24) | 0.020 |
|  | Both | 2.97 (1.02 to 8.64) | 0.046 | 2.49 (0.84 to 7.41) | 0.101 |
| **Social functioning** | | | | | |
| Peer problems at 16 (*n* = 3408) | | | | | |
|  | Depression only | 2.44 (1.39 to 4.29) | 0.002 | 1.92 (1.06 to 3.49) | 0.032 |
|  | PEs only | 1.86 (1.26 to 2.75) | 0.002 | 1.45 (0.96 to 2.19) | 0.075 |
|  | Both | 3.14 (1.44 to 6.81) | 0.004 | 2.48 (1.10 to 5.57) | 0.028 |
| Friends dissatisfaction at 18 (*n* = 2536) | | | | | |
|  | Depression only | 1.54 (0.84 to 2.81) | 0.164 | 1.46 (0.79 to 2.68) | 0.225 |
|  | PEs only | 1.26 (0.82 to 1.92) | 0.294 | 1.20 (0.78 to 1.85) | 0.396 |
|  | Both | 3.04 (1.35 to 6.88) | 0.008 | 2.82 (1.24 to 6.42) | 0.013 |
| **Substance use** | | | | | |
| Harmful drinking at 18 (*n* = 2430) | | | | | |
|  | Depression only | 2.23 (0.94 to 5.29) | 0.069 | 2.19 (0.92 to 5.24) | 0.077 |
|  | PEs only | 1.11 (0.53 to 2.33) | 0.787 | 1.07 (0.51 to 2.27) | 0.854 |
|  | Both | 2.02 (0.47 to 8.63) | 0.344 | 2.02 (0.47 to 8.71) | 0.346 |
| Problem cannabis use at 18 (*n* = 2602) | | | | | |
|  | Depression only | 1.85 (0.78 to 4.35) | 0.161 | 1.77 (0.73 to 4.25) | 0.204 |
|  | PEs only | 0.59 (0.24 to 1.47) | 0.257 | 0.52 (0.21 to 1.30) | 0.160 |
|  | Both | 1.61 (0.38 to 6.85) | 0.516 | 1.83 (0.42 to 7.88) | 0.420 |
| Smoking regularly at 18 (*n* = 2627) | | | | | |
|  | Depression only | 1.30 (0.76 to 2.23) | 0.343 | 1.19 (0.69 to 2.05) | 0.533 |
|  | PEs only | 1.28 (0.90 to 1.83) | 0.168 | 1.17 (0.82 to 1.67) | 0.391 |
|  | Both | 1.74 (0.78 to 3.87) | 0.176 | 1.58 (0.70 to 3.55) | 0.269 |
| Used other drugs at 18 (*n* = 2603) | | | | | |
|  | Depression only | 1.07 (0.56 to 2.04) | 0.830 | 1.11 (0.58 to 2.12) | 0.759 |
|  | PEs only | 1.10 (0.73 to 1.67) | 0.651 | 1.12 (0.74 to 1.71) | 0.591 |
|  | Both | 2.00 (0.86 to 4.63) | 0.107 | 2.22 (0.95 to 5.23) | 0.066 |
| **Illegal and offending behaviour** | | | | | |
| Illegal and offending behaviour by 18 (*n* = 2478) | | | | | |
|  | Depression only | 1.38 (0.79 to 2.41) | 0.255 | 1.50 (0.84 to 2.71) | 0.173 |
|  | PEs only | 1.04 (0.70 to 1.55) | 0.854 | 0.95 (0.62 to 1.43) | 0.791 |
|  | Both | 0.60 (0.18 to 1.97) | 0.395 | 0.71 (0.21 to 2.39) | 0.576 |

^a^Model 1: unadjusted

^b^Model 2: adjusted for gender, social class, housing, maternal education, IQ at 8, total SDQ score at 8

**Table ESM7** Four level analysis of the associations of ‘psychotic experiences only’, ‘depression only’ and ‘both psychotic experiences and depression’ at 18 years (‘neither’ being used as the baseline reference group) with outcomes in early adulthood (18 to 20 years)

|  | | **Model 1^a^** | | **Model 2^b^** | |
| --- | --- | --- | --- | --- | --- |
|  |  | **OR (95% CI)** | **P val** | **OR (95% CI)** | **P val** |
| **Education & employment** | | | | | |
| Did not obtain ≥ 3 A levels A*– C (*n* = 2131) | | | | | |
|  | Depression only | 1.73 (1.33 to 2.26) | <0.001 | 1.70 (1.27 to 2.29) | <0.001 |
|  | PEs only | 1.54 (0.97 to 2.43) | 0.065 | 1.40 (0.84 to 2.34) | 0.198 |
|  | Both | 3.87 (1.87 to 8.02) | <0.001 | 4.45 (2.04 to 9.73) | <0.001 |
| NEET at 20 (*n* = 1861) | | | | | |
|  | Depression only | 1.51 (0.83 to 2.75) | 0.173 | 1.29 (0.70 to 2.37) | 0.423 |
|  | PEs only | 1.36 (0.48 to 3.85) | 0.562 | 1.10 (0.38 to 3.19) | 0.859 |
|  | Both | 5.05 (2.15 to 11.9) | <0.001 | 3.21 (1.30 to 7.91) | 0.011 |
| **Social functioning** | | | | | |
| Friends dissatisfaction at 18 (*n* = 2419) | | | | | |
|  | Depression only | 3.28 (2.41 to 4.47) | <0.001 | 3.26 (2.38 to 4.46) | <0.001 |
|  | PEs only | 1.21 (0.62 to 2.37) | 0.576 | 1.20 (0.61 to 2.36) | 0.599 |
|  | Both | 5.15 (2.92 to 9.07) | <0.001 | 5.16 (2.90 to 9.18) | <0.001 |
| **Substance use** | | | | | |
| Harmful drinking at 18 (*n* = 2308) | | | | | |
|  | Depression only | 1.77 (0.96 to 3.25) | 0.065 | 1.91 (1.03 to 3.54) | 0.040 |
|  | PEs only | 4.80 (2.47 to 9.35) | <0.001 | 5.34 (2.70 to 10.5) | <0.001 |
|  | Both | 5.62 (2.53 to 12.5) | <0.001 | 5.82 (2.58 to 13.1) | <0.001 |
| Problem cannabis use at 18 (*n* = 2475) | | | | | |
|  | Depression only | 3.54 (2.11 to 5.92) | <0.001 | 4.06 (2.38 to 6.91) | <0.001 |
|  | PEs only | 5.67 (2.89 to 11.1) | <0.001 | 6.07 (2.96 to 12.4) | <0.001 |
|  | Both | 11.5 (5.80 to 22.6) | <0.001 | 12.4 (6.05 to 25.5) | <0.001 |
| Smoking regularly at 18 (*n* = 2491) | | | | | |
|  | Depression only | 2.53 (1.91 to 3.37) | <0.001 | 2.40 (1.79 to 3.20) | <0.001 |
|  | PEs only | 2.79 (1.78 to 4.36) | <0.001 | 2.53 (1.60 to 3.99) | <0.001 |
|  | Both | 3.18 (1.83 to 5.52) | <0.001 | 2.87 (1.64 5.02) | <0.001 |
| Used other drugs at 18 (*n* = 2475) | | | | | |
|  | Depression only | 1.87 (1.35 to 2.59) | <0.001 | 2.19 (1.56 to 3.07) | <0.001 |
|  | PEs only | 2.98 (1.87 to 4.75) | <0.001 | 3.36 (2.07 to 5.47) | <0.001 |
|  | Both | 4.23 (2.44 to 7.31) | <0.001 | 5.26 (2.97 to 9.31) | <0.001 |
| **Illegal and offending behaviour** | | | | | |
| Illegal and offending behaviour by 18 (*n* = 2369) | | | | | |
|  | Depression only | 1.21 (0.86 to 1.69) | 0.271 | 1.37 (0.97 to 1.95) | 0.078 |
|  | PEs only | 1.95 (1.19 to 3.19) | 0.008 | 2.30 (1.36 to 3.89) | 0.002 |
|  | Both | 2.21 (1.22 to 4.01) | 0.009 | 2.33 (1.24 to 4.37) | 0.008 |

^a^Model 1: unadjusted

^b^Model 2: adjusted for gender, social class, housing, maternal education, IQ at 8, total SDQ score at 8
