# Supplementary figures and images for: Adverse life outcomes associated with adolescent psychotic experiences and depressive symptoms
Source: Soc Psychiatry Psychiatr Epidemiol. 2018 Mar 19;53(5):497–507. doi: 10.1007/s00127-018-1496-z (PMC5908822; doi:10.1007/s00127-018-1496-z)

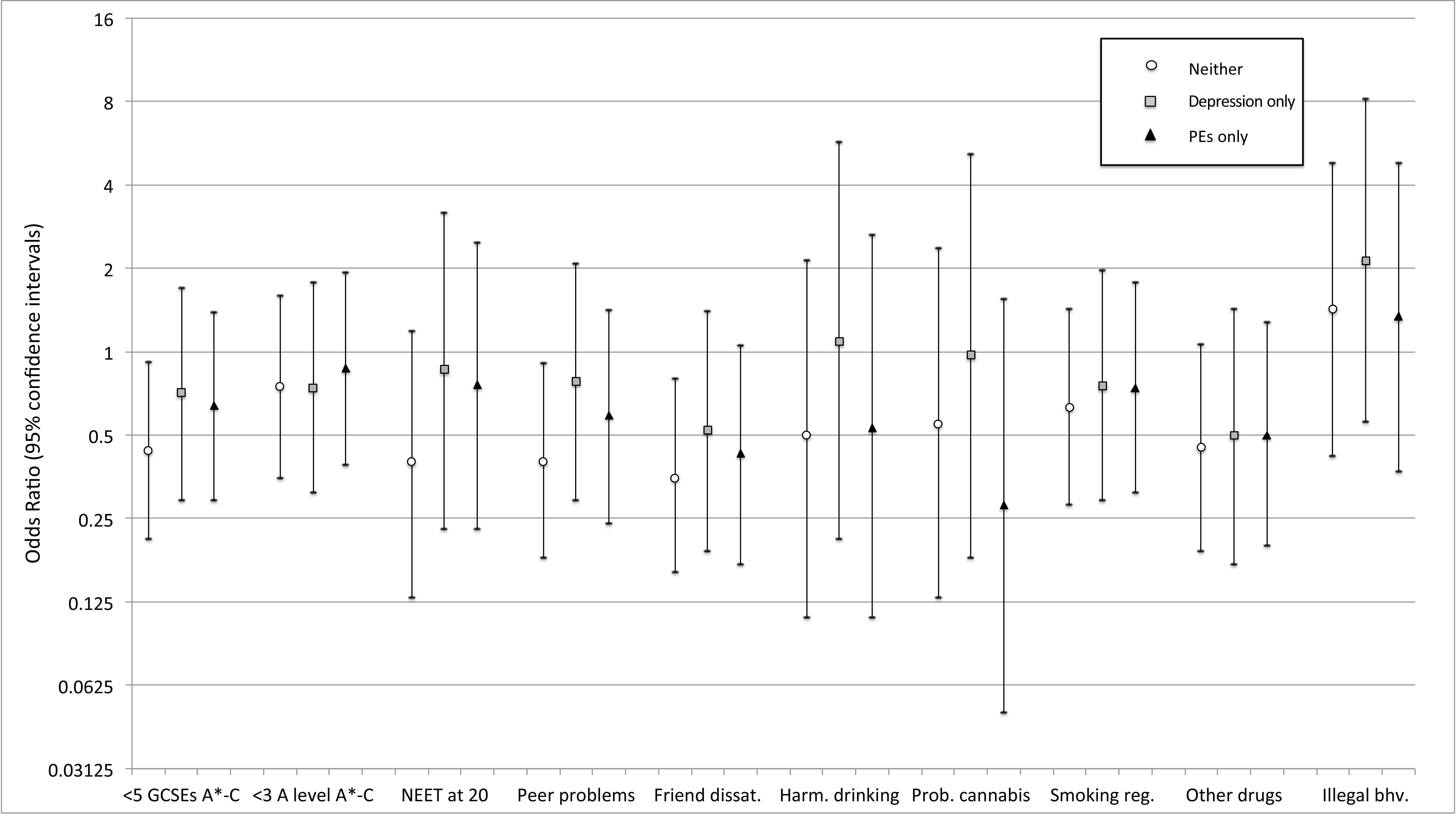

Supplement: Supplementary file 2 — Supplementary material 1 (DOCX 402 KB) [file 127_2018_1496_MOESM2_ESM.tif]
